# Neuromuscular Regeneration: Perspective on the Application of Mesenchymal Stem Cells and Their Secretion Products

**DOI:** 10.1155/2016/9756973

**Published:** 2016-01-06

**Authors:** Ana Rita Caseiro, Tiago Pereira, Galya Ivanova, Ana Lúcia Luís, Ana Colette Maurício

**Affiliations:** ^1^Departamento de Clínicas Veterinárias, Instituto de Ciências Biomédicas de Abel Salazar (ICBAS), Universidade do Porto (UP), Rua de Jorge Viterbo Ferreira, No. 228, 4050-313 Porto, Portugal; ^2^Centro de Estudos de Ciência Animal (CECA), Instituto de Ciências, Tecnologias e Agroambiente da Universidade do Porto (ICETA-UP), Praça Gomes Teixeira, Apartado 55142, 4051-401 Porto, Portugal; ^3^REQUIMTE, Departamento de Química e Bioquímica, Faculdade de Ciências, Universidade do Porto, Rua do Campo Alegre, 4169-007 Porto, Portugal

## Abstract

Mesenchymal stem cells are posing as a promising character in the most recent therapeutic strategies and, since their discovery, extensive knowledge on their features and functions has been gained. In recent years, innovative sources have been disclosed in alternative to the bone marrow, conveying their associated ethical concerns and ease of harvest, such as the umbilical cord tissue and the dental pulp. These are also amenable of cryopreservation and thawing for desired purposes, in benefit of the donor itself or other patients in pressing need. These sources present promising possibilities in becoming useful cell sources for therapeutic applications in the forthcoming years. Effective and potential applications of these cellular-based strategies for the regeneration of peripheral nerve are overviewed, documenting recent advances and identified issues for this research area in the near future. Finally, besides the differentiation capacities attributed to mesenchymal stem cells, advances in the recognition of their effective mode of action in the regenerative theatre have led to a new area of interest: the mesenchymal stem cells' secretome. The paracrine modulatory pathway appears to be a major mechanism by which these are beneficial to nerve regeneration and comprehension on the specific growth factors, cytokine, and extracellular molecules secretion profiles is therefore of great interest.

## 1. Introduction

The peripheral nervous system (PNS) is often involved in severe traumatic events which may result in relevant impairment of occupational and everyday life activities performance. The physical disability itself and the associated painful events limit the quality of life of affected patients [1]. Iatrogenic damage related to surgical procedure is also often observed [2]. When compared to the central nervous system (CNS), the PNS depicts a superior capacity for regeneration, although in severe injuries complete repair is not often observed, and functional recovery is poor [3, 4]. Amongst other factors, this capacity is also dependent on the age of the individual [5], giving the topic additional relevance in an aging world population.

### 1.1. Peripheral Nerve Lesions' Associated Muscular Atrophy (Neurogenic Muscle Atrophy)

Alongside the immediate loss of sensory and voluntary motor functions of the supplied areas and muscle groups, severe nerve injuries are accompanied by atrophy of the latter, resulting from the lack of electrophysiological as well as biochemical communication between the nerve and muscle components [6]. The denervation of a muscle leads to fast progressing muscle mass loss [7, 8], in first instance related to the loss of the contractile machinery, and then to effective loss of muscle fibres, after prolonged, year lasting, denervation periods [7]. The initial events result from unbalanced protein synthesis and proteolysis [9], while the second stage of muscle mass loss results from the combination of cell death and myonuclei apoptosis with decreased satellite cells responsiveness [10].

The general homeostasis and regenerative capacity of skeletal muscle are under significant neural influence. Denervated muscles' fibre type content suffers significant shifts [7], and muscles lose blood supply over time, with significant degeneration of the whole vascular network [11], impairing chances of recovery of muscle function and strength, even if neural function is restored. The regenerative cells pool within the skeletal muscle also seems sensitive to neural control. The loss of this regulation by means of denervation triggers satellite cells function into repetitive proliferative cycles and differentiation [8], ultimately contributing to its exhaustion and long term regenerative impairment of those muscles [10].

The speed of recovery can be further arrested by delayed surgical repair, as occurs in most clinical cases [4]. Accelerated restoration of the nerve structure and function and consequently its electrophysiological stimulatory capacity are key-points for preventing muscle atrophy and promoting functional recovery. The longer nerve communication remains interrupted, the less effective injury activated Schwann cells will be at stimulating regrowth, and the more severe distal stump degeneration will become [12]. The longer a muscle stands devoid of such stimuli the harsher the alterations to its own structure and contractile capacity, and the harder its recovery is upon reestablishment of electrical communication [7].

### 1.2. Peripheral Nerve Injuries and Repair Techniques

In the vast list of diseases affecting the nervous system, and specifically the PNS, traumatic events comprise a relevant source of nerve damage [1]. From crush to sectioning or avulsion, such events severely affect peripheral nerve structure and function, conditioning both sensory and motor transmission pathways.

Focal crush injuries (Sunderland type II), termed axonotmesis injuries, cause disruption of axons and involving myelin sheaths, but the connective support structures are maintained [13, 14]. Recovery from this type of injury does not generally require surgical intervention, and axons regenerate along the preserved endoneural tubes, stimulated by the reactive Schwann cells, ultimately regaining contact with the distal portion of the lesion and finally reinnervating the associated muscle. So, despite being capable of satisfactory self-regeneration, the time-lapse required for the process invariably leads to the atrophy of the formerly supplied muscle groups [15, 16]. Therefore, although no physical reconstruction is necessary for the management of axonotmesis injuries, the development of strategies for an accelerated reconnection process is highly desirable aiming at the optimization of the patient's neuromuscular function recovery.

In the cases of complete nerve severing (Sunderland type V), referred to as neurotmesis injuries, all the nerve fibre structures lose continuity, and spontaneous recuperation becomes more unlikely, demanding important reconstructive microsurgery techniques and, more recently, new therapeutic approaches based on new biomaterials for guiding conduits and cell-based therapies [14, 16, 17].

Despite the extensive effort towards improved surgical techniques for the repair of severely affected nerves, functional recovery indexes on such injuries remain far from desired [18]. While direct suturing of the sectioned nerve endings (neurorrhaphy) would be the most adequate procedure [19], it turns impractical in cases of nerve tissue loss, which require a more complex approach. Nerve grafts can be utilized to bridge this gap in a tension-free manner, from either autologous or allogeneic source [20], as well as other nonnervous tissues, such as blood vessels. However, these grafting techniques have several limitations, including donor site morbidity and donor-recipient nerve diameter and fibre content mismatch, and hence other possibilities were pursued [19–22].

As alternatives to these techniques, nerve conduits from a diversity of biomaterial types and processing techniques started being explored to substitute and overcome the disadvantages of those “organic” options [22]. Materials such as poly(lactic-co-glycolic acid) [23], chitosan [24], collagen [13, 25], and fibrin [26] amongst many others [14, 15, 22] are under study and some have even reached clinical application approval [14, 27, 28].

**Figure 1:**
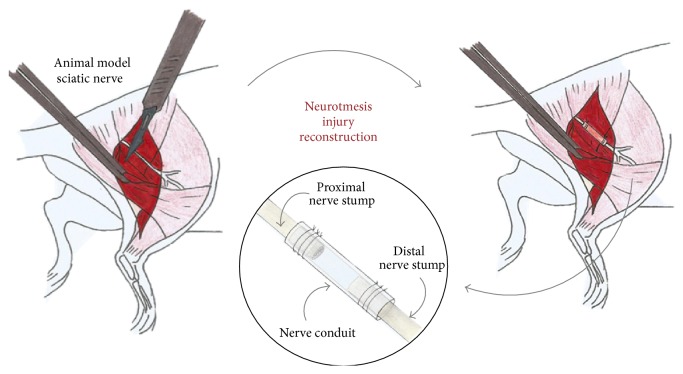
Entubulation or tubulization principle for bridging severe peripheral nerve injuries with loss of nerve tissue, preventing tension-free neurorrhaphy. The sectioned nerve stumps are inserted a certain distance into the conduit and microsutured, in a tension-free manner.

Research on the use of these structures as guiding scaffolds for nerve regrowth, termed entubulation or tubulization (Figure 1) [23, 27], is in candid expansion.

Although the biomaterials alone are capable of supporting, guiding, and even promoting the axonal regeneration and restoration of nerve continuity [29], the effectiveness and speed of the process are still up for improvements, especially for long gaps in the nerve structure [30]. The success of therapies under development will rely on the consideration of a set of issues, namely, the speed of axonal regrowth, the chronic denervation changes in neuromuscular cell populations and surrounding support structures, and the deleterious character of the local microenvironment to the regenerative process [18].

Direct delivery of growth factors is a prospective strategy for enhancing the performance of the nerve conduits and answering these identified issues [22]. There are several techniques for associating these factors to the biomaterial conduit and optimizing their bioactivity and release kinetic [31], but some downsides regarding potential side-effects and interactions jeopardize their application [2]. Another option for functionalizing the nerve conduit is the association of cellular systems, which will provide a wider range of signalling molecules, rather than single selected stimuli. Within the nervous tissue, resident and blood borne cell populations act as bioactive molecules sources in response to injury [32]. Hence, the association of neuro- or glial-derived cells that could act as regenerative triggers in the absence of the native population (i.e., in cases of nerve tissue section/loss, neurotmesis [23]) or as additional boosters to the intrinsic ongoing regenerative process (when no gap exists and direct suture is feasible [25] or neural support structures are preserved, axonotmesis/crush injury[13]) has also been explored. The main difficulty of using these tissue specific cell sources relates to the procedures required for their isolation, expansion, and differentiation, as in the case of Schwann cells [3].

An interesting alternative to the above-mentioned approaches is the usage of mesenchymal stem cells (MSCs) [33], to be further explored herein. MSCs present precious features, turning them highly fitted candidates to cell therapies development: they can be easily expanded, they have the ability to differentiate into different cell types, and they are immune-privileged and immune-modulatory, as well as preferentially homing to injured sites [34]. MSCs have also been recognized as powerful sources of trophic mediators [35, 36], capable of modulating tissues function, including the central [37] and peripheral nervous [38–40], and musculoskeletal systems [41, 42].

Insight into current and prospective sources for MSCs for regenerative medicine approaches is presented ahead, as well as their role as trophic mediators in the neuromuscular regenerative scenery.

## 2. Mesenchymal Stem Cells for Peripheral Nerve Repair and Regeneration

Regenerative medicine and tissue engineering are fast expanding scientific research fields, and MSCs are one of the main entities under study, aiming at the development of innovative therapeutic strategies, covering for a number of diseases, affecting multiple body systems [35]. Mesenchymal stem cells were first described as a specific cell population by Friedenstein's research group in the late 1960s [43–45]. At the time, stem cell populations were thought to reside exclusively in organs with observable regenerative capacity, such as the blood, intestine, bone, and skin. As research progressed, we became aware that they are present in virtually all the body tissues, in variable numbers [46]. These cells generally remain in a quiescent state until activated by significant events, contributing to the efforts of regaining tissue's homeostasis [44, 47, 48].

Through the years, the knowledge on MSCs features and potential has grown exponentially [49, 50] and significant progress has been made towards their better understanding and characterization. In an effort to standardize and unite the scientific community, the Mesenchymal and Tissue Stem Cell Committee, of the International Society for Cellular Therapy (ISCT) gathered a series of recommendations regarding the acceptable criteria for the definition of “mesenchymal stem cell” populations [51]. So, besides their clonogenic and proliferative capacities, while remaining genetically stable and in undifferentiated state, MSCs are also characterized by [51]:

- the plastic adherent ability,
- the absence of definitive hematopoietic lineage markers, such as CD45, CD34, CD14, CD11b, CD79*α*, CD19, and major histocompatibility complex- (MHC-) II/human leukocyte antigen- (HLA-) DR, and expression of nonspecific markers CD105, CD90, and CD73,
- the ability to differentiate into at least three mesodermal lineage cells: osteocytes, chondrocytes, and adipocytes.

Regarding these differentiation abilities [49, 50, 52, 53], MSCs have also been reported to be capable of differentiating into ectodermal and endodermal cell types [54].

### 2.1. Impact of MSCs Transplantation on Host Immune System

The initial notice of MSCs therapeutic potential originated the early concept of “self-therapy” described by Caplan [52], in which immune-rejection was avoided by the possibility of the donor and host of the expanded cells being the same individual. But, unlike terminally differentiated tissue cells, MSCs are practically devoid of HLA-II, a key player in the body's immune response [55, 56], remaining mostly unnoticed as “foreign” elements, when administered to immunocompetent allorecipients [35, 57, 58]. This nonimmunogenic character of MSCs is an essential factor motivating the research field, considering the difficulty of finding matching donors amongst the human population and the challenges of harvesting sufficient numbers of cells from one patient upon necessity [59]. This is also a relevant topic for the progression of research and the development of new therapies, since it allows for the xenogeneic implantation of human-derived cell in appropriate nonimmunosuppressed animal models [16, 17, 39, 60]. This approach provides valuable information on their behaviour and effect on experimental preclinical models that more closely mimic clinical practice reality [58] and aid the translation of therapies to the clinical ground [32].

MSCs were also found to actively impact on immune events [61]. Significant immune-modulatory actions have been attributed to these cells, mediated by secreted inhibitory and stimulatory molecules, as well as through direct cell-to-cell contact [62].

### 2.2. MSCs Sources

The bone marrow is without a doubt the most widely explored source of MSCs for therapeutic purposes [46, 50, 63]. The harvesting procedure is however highly invasive and potentially painful [64], motivating the search for more easily accessible sources. Also, the number [53] and “quality” of the isolate cells strongly depends on the age, gender, and health status of the patient or donor [59].

**Figure 2:**
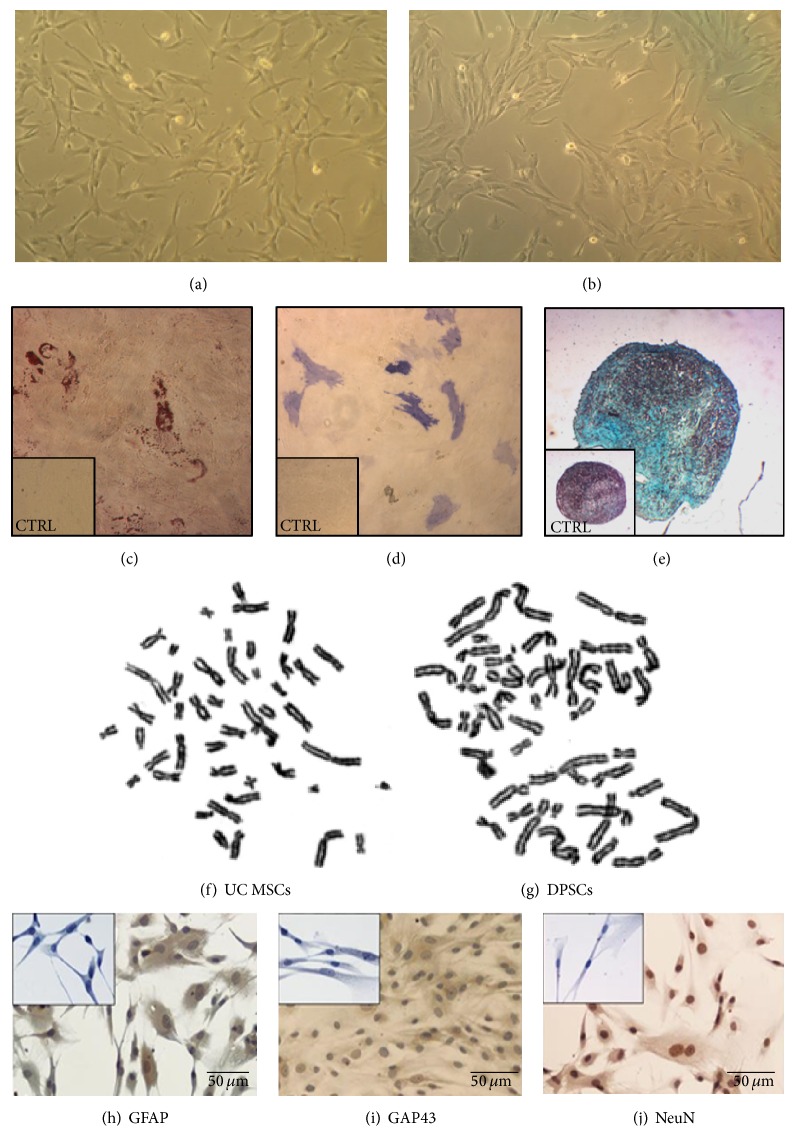
Morphological similarities between DPSCs (a) and UC-MSCs (b) (magnification: 100x), in [41]; qualitative analysis of the tridifferentiation potential of umbilical cord stroma derived MSCs by histological staining methods: adipogenic differentiation [Oil Red O staining (c)]; osteogenic differentiation [Alkaline Phosphatase staining (d)]; and chondrogenic differentiation [Alcian Blue staining (e)], in [16]; UC MSCs (f) and DPSCs (g) karyotype, to assess for chromosomal stability in terms of structure and number of chromosomes and the absence of neoplastic characteristics, demonstrating the stability and safety of the cells in usage; positive immunochemical staining of UC-MSCs for neural markers following* in vitro* culture in neurogenic differentiation medium. Cultured cells stained positive for (h) GFAP which is a glial cell marker; (i) GAP-43 which is related to axonal outgrowth; and (j) NeuN which is a marker for nucleus of neurons. Undifferentiated MSCs cells from Wharton's jelly presenting a negative staining for (small panel inserted in (h)) GFAP; (small panel inserted in (i)) GAP-43, and (small panel inserted in (j)) NeuN (magnification: 200x), in [17].

As alternative to the bone marrow, other sources of MSCs are gaining ground [64], for the minimally invasive nature of their harvest as well as for the lesser ethical concerns surrounding their tissues of origin, such as the umbilical cord blood, adipose tissue (AT-MSCs) [64, 65], or the stromal tissue of the umbilical cord (UC-MSCs) [15, 34, 40] and the dental pulp (DPSCs) (Figure 2) [66, 67].

Cells from these alternative sources display comparable phenotypical features and “stemness” potential [51, 54]. Nonetheless, MSCs from distinct sources are not completely identical, differing in, as an example, their differentiation propensity [64, 68–70] and secretory profiles [71].

## 3. Application of Mesenchymal Stem Cell Based Systems in Neuromuscular Regeneration

Despite all best efforts, available strategies, as discussed in an earlier section, do not seem to meet the healthy nerve's performance, nor the direct end-to-end repair [72], and search towards new methods for improving these outcomes led researchers, including ourselves, to the hypothesis that extraembryonic MSCs would benefit the peripheral nerve regenerative process [40], in both axonotmesis [39, 73] and neurotmesis [16, 17] injury events.

### 3.1. Delivery Routes for Cellular Systems

Distinct routes could be considered for the delivery of the cellular system. Considering that, on occasion, there can be multiple sites of injury, it has been advocated that systemically administered MSCs could selectively home to those sites and aid recovery. Indeed, these authors demonstrated an active role of MSCs on local inflammatory response as well as accelerated functional recovery after peripheral nerve crushing. This approach also promoted enhanced expression of proregenerative factors at the lesion site [74]. This would be a highly practical route, especially when no surgical access to the site is essential. However, it would ultimately require increased numbers of MSCs upon administration, considering the dilution effect on the total body blood volume and, in the case of venous administration, significant entrapment within the pulmonary capillary network. Intramuscular injection of MSCs in the vicinity of a repaired nerve has also been recently proposed [75, 76]. This way, the cellular system would be “within reach” of the damaged nerve and the related muscle groups. Little attention has been given to this option so far. Direct infiltration of the cellular system into the damaged nerve is another viable option, again as long as support structures are maintained or easily reconstructed, but evidence is that the same cellular system bares improved results in association with a biomaterial membrane or conduit [39]. The biomaterial conduit behaves as a structural selective barrier that provides directed guidance to the growing nerve components and protection against fibrotic tissue ingrowth. It shall also allow for the trading of oxygen, growth factors, and metabolites with the surrounding microenvironment while shielding the area from harsh inflammatory events [27]. As such, it presents as a highly rational strategy to combine the advantages of both approaches into hybrid cellular-biomaterial systems, seeking for major advancements in the peripheral nerve regeneration field. It appears as the most appropriate method to maintain the cells in their intended action site. This foreseen concentration effect would in turn lead to an inferior requirement on the administered cell numbers, in comparison to formerly mentioned strategies.

Roughly, there can be considered two methods of associating such systems with the biomaterial, regarding the timing of surgical implantation. Cells can be injected directly into the scaffold upon its implantation [77] or they can be added prior to contact with the damaged nerve. In this latter situation, cells can additionally be allowed to proliferate for variable periods of time, forming adherent monolayers in the interior of the conduit [17, 26, 39].

### 3.2. Effects of MSCs Transplantation on Nerve Regeneration

Several studies have reported beneficial effects of MSCs' systems on nerve injury models (Table 1) that can be overall listed as follows: (i) modulation of the inflammatory environment on the site; (ii) modulation of the Wallerian degeneration stage; (iii) increased thickness of the myelin sheaths; (iv) accelerated fibre regeneration and in increased numbers; (v) improved fibre organization; (vi) enhanced vascularization of the regenerating site; and (vii) reduction of fibrotic scaring.

The specific mechanisms by which MSCs impact on peripheral nerve regeneration still care irrefutable evidence, but scientific community strongly leans towards paracrine modulation role, on basis of a number of observations. MSCs can be identified near the injury site, generally in association with the* vasa nervora* [95], but not generally penetrating the regenerating nerve, although their presence relates to stronger expression of Schwann and neuronal cell markers on the nerve itself [78]. Indeed MSCs colocalize with increased neurotrophic expression, demonstrating their role as paracrine stimulators for the resident population [89]. Therefore, evidence suggests that they may mostly act directly on the Schwann cells population, increasing the expression of myelinisation and Schwann cells activity-related genes and trophic factors production [89, 95], depicted by increased myelin sheath thickness, therefore modulating the events of Wallerian degeneration and axonal regeneration [16, 39]. Parallel to direct stimulatory effects on Schwann cells (boosting their own expression of neuromodulatory factors, e.g., CNTF and GDNF [95] that act on axonal growth), the array of molecules provided by MSCs includes strong angiogenic promoters, which are associated with improved outcomes, as detailed ahead. The antifibrotic effect attributed to MSCs is also described in peripheral nerve studies [16, 78]. This effect is possibly associated with modulation of the immune response exerted by the MSCs on active inflammatory sites [62] and in association with biomaterial implants [42], as observed for other tissues and applications. Besides the effects on Schwann cells and axonal growth on the site of injury, the association of MSCs with therapeutic reconstruction has also been demonstrated to protect from neuronal cell death, associated with the axonal retrograde transportation of neurotrophic factors [82].

The need for predifferentiation of delivered MSCs towards neuron related phenotypes (Figures 2(h), 2(i), and 2(j)) is also still unanswered [4, 39]. On one hand, the differentiation process seems to boost neurotrophins secretion [38, 96, 97]. This observation is dominantly supported by* in vivo* data but, on the other hand, controversial reports can also be found in the available literature (references cited in Table 1). Also the process of predifferentiation is time-consuming, affecting the availability of cellular therapies for prompt and speedy reconstructions of affected nerves [80]. It is still not clear whether the differentiation process interferes with postimplantation survival and thus with the window of action of the system [4]. The effects of exogenous manipulation on cells' viability and features and the variations in applied protocols and biomaterials might reason the lack of consensus on the matter. Also, as no definitive data is available, it should be left under consideration that differentiated MSCs may affect neuromuscular regeneration in a more targeted way, while undifferentiated MSCs may play a greater role in supportive functions, such as neoangiogenesis and inflammatory modulation.

### 3.3. Association of MSCs with Biomaterial Nerve Conduits

After peripheral nerve crush, the deposition of AT-MSCs-biomaterial system onto the nerve lesion accelerated both sensory and function recovery [38]. Alternatively, the crushed nerve can be wrapped around by the system in a membrane form [39, 73]. Amniotic fluid derived- (AF-) MSCs promoted function recovery, as wells as Schwann cells activity, and limited fibrosis on site following envelopment with a biomaterial membrane [78].

In complete section situations, even when the damaged nerve is amenable of tension-free suturing, the association of a UC-MSCs system (by local deposition) with the surgical therapy has proved advantageous to the overall regenerative process. Modulation of the Wallerian degeneration phase and improved fibre organization could be attributed to MSCs' action, and thicker myelin sheaths as well as increased numbers of regenerating fibres could be observed for the long term recovery, correlating with positive function recovery indicators [16].

Entubulation or complete wrapping with a MSCs-biomaterial system can also benefit direct end-to-end repair, as well as in graft repaired nerves. Biomaterial conduits associated with undifferentiated and neuroglial differentiated UC-MSCs bared slight contributions to motor deficit recovery. At a histological level, both cell systems seemed to improve microfasciculation observed in surgically reconstructed groups [17].

In patent nerve gaps, AT- and BM-MSCs-loaded conduits contribute to an accelerated kick-off of the regenerative response, promoting enhanced axonal ingrowth into the conduit in the first weeks after surgery [26, 80, 85], almost reaching the effectiveness of Schwann cells-loaded ones [26]. To a longer term, BM-MSCs-loaded conduits also resulted in regenerating nerves with overall superior organization and vascularization, and significant increase in the number of myelinated nerve fibres, and thickness of the myelin sheaths [3, 77], correlating with improved functional indexes [77]. AF-MSCs systems also ensured bridging of the nerve gap with properly oriented, larger axonal fibres and diminished surrounding inflammation, accompanied by regain of electrophysiological conduction and motor function [79]. More recently, Georgiou et al. functionalized a commercially available conduit with an inner type I collagen membrane with self-aligned glial-like differentiated AT-MSCs, and although no function indicators are presented, these approaches granted a 3,5-fold increase in the axons reaching the distal stump and increased myelin sheath thickness, comparing to the conduit alone [83]. Along the UC-, BM-, and AT-MSCs detailed herein, many other examples and applications can be found in hybrid cellular-biomaterial systems, such as skin-derived MSCs [28]. DPSCs have also been suggested to elicit superior regenerative response in tubularized nerve injuries [91, 92]. DPSCs granted early connection of regenerating fibres to the distal nerve stump (100% versus 50% of untreated controls, after only 5 days) [93].

Considering the electrophysiology of the neuromuscular tissue, and the recognition that it influences its homeostasis and regeneration, electrofunctionalised biomaterials may also play a relevant role. Low frequency stimulation implemented shortly after injury seems to promote improved electrophysiological recovery and myelinated fibres content in reconstructed nerves [98, 99]. Nerve conduits functionalized with electroconductive agents, such as carbon nanotubes and polypyrrole, boosted axon regeneration and neuromuscular recovery in a neurotmesis lesion with tissue loss, achieving comparable results to end-to-end repaired nerves. The actions of the nanotubes seemed further promoted by the MSCs association, in terms of nerve regeneration indexes and functional assessment [94]. The observed effects may relate to the direct effect on nerve structures stimulation, as well as to their influence on the grafted cells activity. Carbon nanotubes are capable of modulating MSCs activity, enhancing the expression of neuronal markers and selected neurotrophic growth factors (NGF and BDNF) in cultured BM-MSCs [100].

These and other examples of MSCs-based systems intended for PNS lesions regeneration are summarized in Table 1.

Evidence supports that the addition of either differentiated or undifferentiated MSCs to regenerative medicine reconstructive approaches boosts healing through structural and functional responses in relevant animal models, opening the possibilities for the translation of such experimental therapies for clinical practice. Regarding animal models, all of the above-mentioned studies utilised rodents as experimental subjects. However, the proportion and effective size of the lesions observed do not exactly replicate the clinical reality in human patients. Further advancement of these trialled therapies will certainly require the scale-up to larger models, approaching the clinical settings, such as the ovine model proposed by Casañas et al. [101].

**Figure 3:**
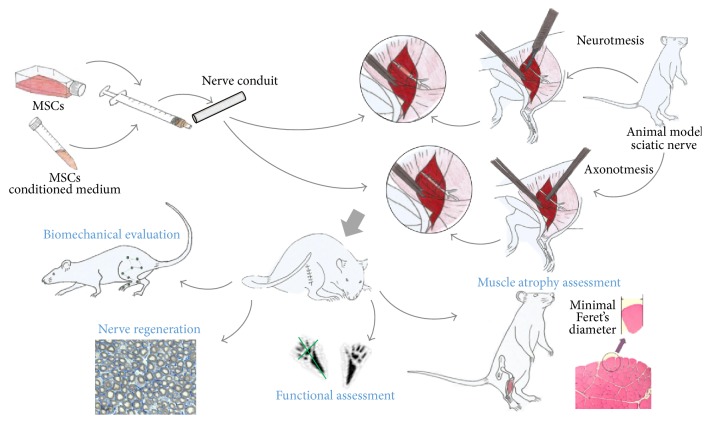
Proposed strategies for* in vivo* preclinical assessment of MSCs and MSCs' CM-based strategies for PNS regeneration in axonotmesis and neurotmesis injuries.

So, in neuromuscular regeneration, stem cells' major action is possibly through their fine secretory activities, exerting paracrine modulatory actions in the target tissue. Consequently, some groups investigated the extent to which the presence of the cells themselves was absolutely essential to the observation of beneficial effects (Figure 3). Some results demonstrate that the application of the secretion products alone (in the form of conditioned medium (CM)) displayed similar if not improved results, as demonstrated for volumetric skeletal muscle injuries [42], central nervous system [102], skin wounds [103], fulminating hepatic failure [104] and hepatic transplant [105], chronic kidney disease [106], and acute lung injury [107, 108].

### 3.4. Impact of MSCs Therapy on Regional Muscle Atrophy

Bearing in mind the importance of the muscular component for full function regain, strategic actions towards the prevention of it atrophy and/or its speeded recovery should also be considered, as it has been demonstrated that AT-MSCs differentiated towards Schwann phenotype positively affect muscular activity restoration after sectioned nerve repair [76]. Neurosphere induced AT-MSCs are also described to provide increased muscle fibre diameter and muscle weight comparing to empty nerve conduits [81].

BM-MSCs systemic (IV), regional (IM) [75], or biomaterial associated delivery [77] also depicted positive results in terms of counteracting neurogenic atrophy. The MSCs-biomaterial system was also associated with diminished decrease of creatine phosphokinase levels in muscle, as well as improving functional recovery in mice [77].

Although the specific evaluation of skeletal muscle macroscopic and microscopic features is not always employed, significant information on its status can be taken from their performance, more frequently assessed in PNS studies through functional testing, as has been highlighted throughout the previous sections.

## 4. Mesenchymal Stem Cell's Secretome: The Potential for Neuromuscular Regeneration

Earlier in the application of MSCs as therapeutic agents, they were hypothesized to contribute to the healing process by effectively differentiating into the required cell types and replacing the damaged cells in their functions. However, as research progressed, evidence arose in that this mode of action might not be universal to every body tissue. It was observed that in some instances MSCs remained in their undifferentiated state at the lesion site or in its vicinity [78], or that could only be identified for a short period of time, or even that only minimal percentages of the MSCs would effectively differentiate and integrate host tissues [63, 80]. Nevertheless, better outcomes were consistently observed whenever MSCs were added to the therapeutic system.

Although MSCs can acquire neuron-like appearance and phenotypical profiles and even display electrical activity under specific inducing stimuli, effective differentiation into active neuronal cells is yet to be demonstrated [63]. As previously mentioned, these observations further support the belief that MSCs benefits to tissue regeneration reside in mechanisms alternative to differentiation and were attributed to the secretion products of those MSCs [17, 78]. In recent years, research has focused on deepening the knowledge on the effective composition of the MSCs secretome. A wide range of growth factors, cytokines, chemokines, and extracellular matrix components have already been identified [36, 109–111]; many of them are known to impact on neuromuscular tissues structure, function, and regeneration.

### 4.1. The Role of Growth Factors in Neuromuscular Regeneration

Peripheral nerve injury triggers a complex cascade of events, as detailed elsewhere [2, 32], where the glial population of Schwann cells assume a primary role, at a structural level, but also as major paracrine modulators, through the secretion of key bioactive factor that will determine axonal regeneration.

CNS neuroprotection and neuroregeneration functions have been attributed to a group of growth factors, such as nerve growth factor (NGF), brain-derived neurotrophic factor (BDNF), neurotrophin- (NT-) 3, NT-4/5, ciliary neurotrophic factor (CNTF), basic fibroblast growth factor (bFGF), and erythropoietin (EPO) [112]. A similar arrangement of growth factors seems to modulate peripheral nerve growth and regeneration [32, 78], with additional relevance to vascular endothelial growth factor (VEGF) and insulin-like growth factor- (IGF-) 1 [31, 113].

Neurotrophic factors can be classified into three major groups, regarding their targeted receptors, as neurotrophins (NGF, BDNF, NT-3, and NT-4/5), neurokines (CNTF and leukemia inhibitory factor (LIF)), and the transforming growth factor- (TGF-) *β* family (TGF-*β*1, TGF-*β*2, TGF-*β*3, and glial cell-derived neurotrophic factor (GDNF)) [78, 114]. Through diverse pathways, these molecules majorly act in guiding axonal growth, in apoptosis prevention, and in promoting Schwann cells activity [38, 114]. Schwann cells action modulation and potentiation might come as one of the main actions of these targeted factors [78], since axonal growth and differential sensory and motor neurons development strongly depend on the regulatory “cocktail” to which they are exposed, which is* in situ* provided by the Schwann cells population [78, 113].

Some specific roles and functions have already been identified for many of these growth factors, enlightening on the mechanisms by which MSCs specifically contribute to the observed benefits in neuromuscular regeneration. These and other factors directly impact on axonal extension but the observed effects upon application of MSCs are assumingly related to their parallel action on the glial population. This results in the amplification of delivered factors' effect by the intrinsic regenerative cocktail provided by the Schwann cells. Detailed molecular pathways analysis on axonal growth and growth factor interaction [114–116] escapes the scope of the present review, but some relevant observations on the impact of key factors are commented herein.

BDNF assumes an important role since its ablation from the MSCs/biomaterial system significantly impairs nerve length [38]. This event naturally occurs in chronic injuries due to a decreased production by endogenous cells. However, its benefit is dose-dependent and may become deleterious for axonal growth in high concentration [117].

IGF-1 seems to exert relevant protection against apoptosis in CNS populations [118], and similar neuroprotection and supportive functions are associated with the PNS.

NGF's importance against neuronal apoptosis is debatable, since its neutralization does not significantly affect neuroprotective actions [118]. However, for PNS, NGF delivery in different situations seems to improve myelinization and neural following complete transection and reconstruction [31].

CNTF and NT-3 are overexpressed in MSCs treated nerves and correlate with improved structural and functional findings [78]. Besides its attributed neuroprotective actions, CNTF has also been recognized as myotrophic, contributing to the attenuation of the morphological and functional changes resultant from skeletal muscle denervation [6]. Despite their individual functions, the key to successful neuroregeneration is in their synergistic action. The combined actions of NGF, CNTF, and GDNF were proven to override their individual benefits in nervous function recovery [119].

A neurotrophic and neuroprotective effect has also been attributed to cerebral dopamine neurotrophic factor (CDNF) in CNS and PNS injuries. It inhibits the production of proinflammatory cytokines and promotes nerve regrowth after sectioning and function recuperation, as demonstrated by the administration of CDNF enhanced MSCs (transfected). This growth factor also exerted a positive influence on the muscle atrophy associated with the nerve lesion [120].

VEGFs also assumes a preponderant role in peripheral nerve regeneration, although seemingly to be in an indirect manner, by boosting the proliferation of vascular cells which are in turn additional sources of neurotrophins [38]. Impaired VEGF expression in response to injury, as it occurs in aged individuals, appears to relate to poorer outcomes in peripheral nerve regeneration [5]. The strict relationship between vascular and neural events is evident in almost any body tissue, and such structures are commonly referred to as “neurovascular” units. Furthermore, the loss of the vascular supply to denervated muscles and consequent hypoxic and underperfused environment is deemed to be a significant factor impairing its recovery after long term denervation [11]. Hence, VEGF is crucial to both the reinnervation process and the recovery from the associated skeletal muscle atrophy.

Additional detail and summarized evidence on the importance of these and other neurotrophic factors for peripheral nerve regeneration can be found in [22]. Many other neurotrophins and neuroregulins are known to be expressed by MSCs, as well as molecules related to extracellular matrix (ECM) components, neurite guidance, and myelinization, possibly impacting on MSCs actions at lesion sites [38].

### 4.2. Neurotrophic Factors Secretion by MSCs

Several publications are dedicated to the detailed analysis of the composition and methods of analysis of the bioactive factors composition of diverse MSCs populations [36, 109, 110, 121–123]. Interestingly, one work reported that the major part of molecules secreted by BM-MSCs accounts for ECM components, while only minor proportions include growth factors and inflammatory regulators [124]. Noteworthy, some of these secreted bioactive factors are not only found in soluble forms, but rather associated with exocytic vesicles (exosomes) transporting additional agents and genetic data (mRNA and miRNA) to the system [125–127]. Exosomes have been demonstrated to interact with neural cells populations [128] and promote neurite outgrowth* in vitro* [127].

Most of the neurotrophic factor previously detailed have been associated with MSCs sources applied in cellular or MSCs-biomaterial therapeutic strategies (Table 2).

MSCs from the UC and the BM are described to secrete significant levels of several well-described proliferative, chemotactic, and immune-modulatory molecules, such as TGF-*β*, G-colony stimulating factor (CSF), GM-CSF, monocyte chemotactic protein- (MCP-) 1, interleukin- (IL-) 6 and IL-8 [111, 129]. Other major vasculogenic factors have also been detected in MSCs populations, and BM- and AF-MSCs depict interesting values of these, in addition to proliferative and chemotactic ones [103, 130].

BM-MSCs, AT-MSCs, and to a greater extent UC-MSCs have all been demonstrated capable of secreting significant neuroregulatory factors to its surrounding medium, such as bFGF, NGF, NT-3, NT-4, and GDNF [70]. Differentiation of UC-MSCs towards glial phenotypes seems to further enhance the already interesting basal undifferentiated state secretion levels of BDNF, NGF, and NT-3 [97]. Undifferentiated DPSCs express interesting amounts of bFGF, TGF-*β*s, and VEGF [71], and also specific neurotrophic factors, such as BDNF, NGF, NT-3, and GDNF [96].

Although one could consider that these molecules are reportedly secreted in small amounts, it is essential to notice that the therapeutic doses of growth factors in a delivery system generally stand at considerable low ranges, from nano- to microgram amounts [31].

From the analysis of the available literature, there seems to be little agreement on exact content of the MSCs secretome [62]. A number of factors that can be responsible for these differences, such as the different assays employed and their respective sensitivities, the tissue source from where the MSCs were harvested, as well as the standard and conditioning culture conditions to which the MSCs are subjected [62, 109, 123]. Another important observation is that conditioning time influences the content and quantity of present factors and other proteins [132, 137], as does the passage number of the cultured cells [132].

Those last points are worth some consideration since, beyond modulating their surrounding environment, MSCs are sensitive themselves to signalling factors, altering their secretory profile in response to their surroundings [62] which can be explored to our advantage, modulating the secretion activities of the MSCs to better fit the therapeutic intents. Modulating agents can be, as an example, the presence of inflammatory cytokines and other stimuli [138–140], growth factors medium supplementation [139, 141, 142], or reduced oxygen tension [38, 103, 107].

Hypoxic environments during conditioning seem to be a determining factor in enhancing provasculogenic factors secretion by MSCs populations [103, 131, 143], as well as neuroprotective/neuroregenerative factors expression, such as NGF and BDNF [38]. The induction of MSCs towards phenotypes through differentiation medium [38, 96, 139] is also an interesting tool to boost target growth factor secretion, such as BDNF [38], NGF, NT-3, and GDNF [96]. Neurotrophic factors secretion can also be boosted by EGF/bFGF stimulation during expansion culture [142].

Much more emphasis has been given to this cellular product on CNS models and applications [37, 112, 132, 137, 144], rather than to PNS. Paring up the contents of MSCs secretome and the biomolecular players of neuromuscular regeneration and considering that the native developmental and regenerative environment of neural tissues involves multiple growth factors, in diverse and dynamic combinations, the potential benefits of the association of MSCs and their products to nerve conduits (Figure 3) and other therapies turn evident [96].

Besides the obviously essential role of the secreted growth factor fraction of MSCs conditioned medium, other components can be associated to vehicles, such as a great variety and amount of metabolic substrates, as demonstrated in [111], aiding the synthetic activities of delivered and native cells. Amongst other metabolite fractions, amino acids assume a preponderant role, providing basic molecules for further growth factors production by active cell populations.

MSCs' products, and more specifically MSCs' CM, may rise as a relevant tool for the regenerative medicine tool. It would grant the ready availability of biomolecule cocktails in therapeutic dosages from a small number of cells, amenable of preservation (frozen or lyophilized) for their timely application, the factors' concentration, and even content manipulation. By associating this with appropriate biomaterials, sustained delivery could be achieved and assure proregenerative environments on neuromuscular injuries, speeding up and optimizing their recovery.

## 5. Conclusions

The present review summarises the vision on why and how MSCs have been contributing and may hereafter aid the search for improved therapy methods for neuromuscular regeneration. MSCs present interesting features for the task, and similar characteristics have been attributed to other recently appealing sources, such as the umbilical cord stroma and the dental pulp. These cell populations may become privileged sources of stem cells for multiple therapeutic purposes, given their accessibility and ease of banking.

Some other issues rather than the cell source itself still require thorough addressing, in search for optimised strategies. Which route is more suited for the administration of MSCs' systems? Which would be the most suitable biomaterials used as nerve conduits for optimizing stem cells' transplants effectiveness? Is it more advantageous to induce them toward neural lineages prior to application or should we opt for undifferentiated cells? How does the local neuromuscular environment affect delivered MSCs performance and action?

As literature demonstrates, MSCs contribute to improved axonal ingrowth and myelinization, resulting in electrophysiological and functional advantage over conventional grafting strategies and biomaterial nerve conduits alone. Further, considering the dominant trophic function attributed to these cells, details on the current knowledge of the MSCs secretory capacities and their secretome characterization are analysed, sustaining its potential as a new interesting strategy to explore. Successful application of such therapies will bring tremendous advantages in terms of therapy availability, expansion, and cell numbers requirement and also the controlled administration of “naturally” occurring growth factors cocktails. As observed for other tissue systems, we propose that MSCs cultures conditioned medium can turn up as a mode of delivery of proregenerative biomolecules to injured peripheral nerves, in search for the potentiation of regenerative medicine approaches.

## Figures and Tables

**Table 1:** Examples of MSCs systems applications in peripheral nerve injury models.

	Cellular system	MSC's differentiation status	Delivery mode	References
Axonotmesis lesions	rAF-MSCs	Undifferentiated	Fibrin glue + cellulose derivate membrane	[78]
	mAT-MSCs	Undifferentiated	Matrigel	[38]
	hAT-MSCs	Undifferentiated	Systemic intravenous delivery	[74]
	hUC-MSCs	Undifferentiated	Local injection/chitosan III membranes	[73]
	hUC-MSCs	Undifferentiated & neuroglial differentiated	Poly(DL-lactide-e-caprolactone) (PLC) membranes	[39]

Neurotmesis lesions	hAF-MSCs	Undifferentiated	Fibrin glue + cellulose derivate membrane	[79]
	rAT-MSCs	Undifferentiated	Fibrin + PHB (poly-3-hydroxybutyrate) conduit	[80]
	rAT-MSCs	Neuroglial differentiated	Fibrin conduit	[26]
	hAT-MSCs	Neuroglial differentiated	Chitosan coated silicon tube/silicon tube	[81]
	rAT-MSCs	Neuroglial differentiated	PCL (polycaprolactone) conduit	[82]
	rAT-MSCs	Neuroglial differentiated	Tethered type 1 collagen gel + collagen membrane	[83]
	rAT-MSCs	Neuroglial differentiated	Collagen gel + xenogeneic acellular nerve matrix	[84]
	hAT-MSCs	Undifferentiated & growth factor stimulated	Fibrin conduit	[85]
	hAT-MSCs	Undifferentiated & neuroglial differentiated	Fibrin conduit	[86]
	rAT-MSCs	Undifferentiated & neuroglial differentiated	Fibrin conduit	[86]
	hAT-MSCs	Undifferentiated & neuroglial differentiated	Silastic conduit	[87]
	rAT-MSCs	Undifferentiated & neuroglial differentiated	Local intramuscular delivery	[76]
	mBM-MSCs	Undifferentiated	Resorbable collagen conduit	[3]
	rBM-MSCs	Undifferentiated	Laminin-modified chitosan + silicon conduit	[88]
	mBM-MSCs	Undifferentiated	PCL (polycaprolactone) conduit	[77]
	rBM-MSCs	Undifferentiated	Fibrin + PCL (polycaprolactone) conduit	[89]
	rBM-MSCs	Undifferentiated	Systemic intravenous or local intramuscular delivery	[75]
	rBM-MSCs	Neuroglial differentiated	Fibrin conduit	[26]
	rBM-MSCs	Undifferentiated & neuroglial differentiated	Matrigel graft	[90]
	rDP-MSCs	Undifferentiated	Type 1 collagen gel + silicone conduit	[91]
	rDP-MSCs	Undifferentiated	Type 1 collagen gel + silicone conduit	[92]
	rDP-MSCs	Undifferentiated	Type 1 collagen gel + poly-DL-lactide-co-glycolide (PLGA) conduit	[93]
	hUC-MSCs	Undifferentiated	Injected/gelatin-thrombin matrix	[16]
	hUC-MSCs	Undifferentiated	Polyvinyl alcohol (PVA), PVA-carbon nanotubes (CNTs) conduits	[94]
	hUC-MSCs	Undifferentiated & neuroglial differentiated	Poly(DL-lactide-epsilon-caprolactone) (PLC) membranes	[17]

h (human), m (mouse), r (rat); AF (amniotic fluid), AT (adipose tissue), BM (bone marrow), DP (dental pulp), and UC (umbilical cord tissue) derived MSCs; neuroglial differentiation includes the application of differentiation/induction protocol towards neuronal or glial phenotypes.

**Table 2:** Secretion of major neurotrophic and support factors by MSCs from different tissue sources.

	BDNF	bFGF	GDNF	IGF	NGF	NT-3	NT-4/5	VEGF
AF	•	°	•	•	•	•	•	•
AT	•	°	°	•	•	•	•	•
BM	•	°	•	•	°	•	•	•
DP	•	•	•	•	•	•	•	•
UC	•	•	•	—	•	•	•	•

AF (amniotic fluid), AT (adipose tissue), BM (bone marrow), DP (dental pulp), and UC (umbilical cord tissue) derived MSCs; •: detected in cell culture supernatant/conditioned medium; °: conflicting reports on the detection of the specific factor in cell culture supernatant/conditioned medium; —: not disclosed in presented literature; and references: [38, 70, 71, 74, 78, 96, 97, 103, 111, 131–136].

## Abbreviations

AF-MSCs: Amniotic fluid derived mesenchymal stem cells
AT-MSCs: Adipose tissue derived mesenchymal stem cells
BDNF: Brain-derived neurotrophic factor
bFGF: Basic fibroblast growth factors or fibroblast growth factors -2
BM-MSCs: Bone marrow derived mesenchymal stem cells
CDNF: Cerebral dopamine neurotrophic factor
CM: Condition medium
CNS: Central nervous system
CNTF: Ciliary neurotrophic factor
CSF: Colony stimulating factor
DPSCs: Dental pulp derived stem cells
ECM: Extracellular matrix
EPO: Erythropoietin
GDNF: Glial cell-derived neurotrophic factor
HLA: Human leukocyte antigen
IGF: Insulin-like growth factor
IL: Interleukin
ISCT: International Society for Cellular Therapy
LIF: Leukemia inhibitory factor
LPS: Lipopolysaccharide
MCP: Monocyte chemotactic protein 1/chemokine (C-C motif) ligand 2 (CCL2)
MHC: Major histocompatibility complex
MSCs: Mesenchymal stem cells
NGF: Nerve growth factor
NT: Neurotrophin
PNS: Peripheral nervous system
TGF: Transforming growth factor
TGF: Transforming growth factor
TNF: Tumoral necrosis factor
UC-MSCs: Umbilical cord stroma/Wharton's jelly derived mesenchymal stem cells
VEGF: Vascular endothelial growth factor.
